# Intra-articular triamcinolone hexacetonide injections in hands osteoarthritis ‒ A double-blinded randomized controlled trial with a one year follow-up

**DOI:** 10.1016/j.clinsp.2022.100036

**Published:** 2022-07-26

**Authors:** Natalia de Oliva Spolidoro Paschoal, Jamil Natour, Flavia Soares Machado, Hilda Alcântara Veiga de Oliveira, Artur da Rocha Correa Fernandes, Rita Nely Vilar Furtado

**Affiliations:** Universidade Federal de São Paulo (UNIFESP), São Paulo, SP, Brazil

**Keywords:** Osteoarthritis, Intra-articular injection, Corticosteroid, Hands

## Abstract

**Objective:**

to evaluate the effectiveness of triamcinolone Hexacetonide (TH) Intra-Articular Injection (IAI) in hand Interphalangeal Joints (IP) of Osteoarthritis (OA) patients to improve pain and joint swelling; improve function, goniometry, and grasping force, and assess IAI influence on radiographic evolution over 1-year.

**Methods:**

A randomized, double-blind study. 60 patients who underwent IAI at the most symptomatic IP joint were randomly assigned to receive TH+Lidocaine (LD) (TH/LD group) or just LD (LD group). Patients were assessed blindly for 1-year, at baseline and 1, 4, 8, 12, and 48 weeks. The following variables were assessed: articular pain and swelling, AUSCAN and COCHIN functional questionnaires, grip and pinch strength, goniometry, perception of improvement, acetaminophen consumption, and simple radiography. Repeated-measures ANOVA test was used to analyze the intervention response.

**Results:**

Sixty patients completed the follow-up. There were nine missed assessments. 97% were women; mean age of 61-years (SD = 8.2), and approximately 5-years of disease (SD = 3.6). Half of the patients present radiographic classification Kellgren and Lawrence (KL) grades I and II, and the other half grades III and IV. The two groups evolved similarly at 48-weeks. TH/LD group had a better evaluation in joint swelling and acetaminophen consumption (p = 0.04 and p < 0.001, respectively) at 48-weeks. Radiographically there was no statistical difference between groups (KL, p = 0.564; erosive OA, p = 0.999; worsening, p = 0.573).

**Conclusion:**

The IAI IP hands OA is effective for the improvement of joint swelling and decrease of analgesic consumption and does not influence the radiographic evolution of the injected joint.

## Highlights

• Hands osteoarthritis treatment.
• Intra-articular corticosteroid injection.

## Introduction

Osteoarthritis (OA) is the most prevalent form of joint disease in the world. The primary symptoms of OA are pain, loss of function mobility, and reduced quality of life.^1^, ^2^, ^3^

OA of the hands with Interphalangeal Joint (IP) involvement is one of the most characteristic subtypes of the disease, but there are few studies evaluating the effectiveness of the immense diversity of therapies available for hand OA. The number of studies has been growing in recent years, but differences in sample size, patient types, study design, type of intervention, and type of joints involved make it difficult to offer reliable, practical recommendations for appropriate therapy in these patients.^2^,^4^ Several clinical trials and systematic reviews support the effectiveness and safety of Intra Articular Injection (IAI) with corticosteroids in knee OA. Its effectiveness is rapid, with peak action occurring in less than a week.^5^, ^6^, ^7^, ^8^, ^9^

In patients with OA of the IP, IAI with corticosteroid was shown to be effective for the improvement of pain on movement and joint swelling at 12-weeks of follow-up.^10^ Despite the potential to improve local symptoms, the authors did not know whether this procedure would influence joint pain, swelling, range of motion, or function in these patients in longer follow-up. The authors also did not find controlled studies assessing the effect of this procedure in the interphalangeal joint according to the radiographic evolution, either in the short or long term.

A recent systematic review concluded that the IAI with corticosteroids in the first carpometacarpal is no more effective than placebo but is effective in the IP.^1^

The primary objective of the present study was to compare, at 48-weeks of follow-up, the effectiveness of an IAI with Triamcinolone Hexacetonide (TH) and lidocaine versus an IAI with only lidocaine in the most symptomatic IP hand joint of patients with primary OA in improving pain and joint swelling. As a secondary objective, the authors compared these two groups according to the function, goniometry, grip strength, analgesic use, and radiographic evolution in the injected joint.

## Methods

### Study design

This study was a randomized, controlled, double-blind study featuring a 1-year follow-up.

### Sample

The 60 patients were recruited from the outpatient clinics of the Universidade Federal de São Paulo – UNIFESP, São Paulo, Brazil, from August 2011 to August 2013.

The present study was approved by the local ethics committee (CEP 0956/11), and all recruited patients provided their written and informed consent. This study was registered with ClinicalTrials.gov (NCT02102620).

The patients had to fulfill the following inclusion criteria: have a diagnosis of primary hand OA with Proximal IP (PIP) or Distal IP (DIP) involvement, as based on the criteria specified by the American College of Rheumatology 1990 for hand osteoarthritis; be 40-years of age or older; and have pain in at least one IP hand joint between 3 cm and 8 cm according to the Visual Analogue Scale of Resting Pain (VASr: 0–10 cm).

The exclusion criteria were as follows: patients with changes in the use of oral corticosteroids and non-steroidal anti-inflammatory drugs in the last 30-days; patients with changes in drug use or other treatments for OA (rehabilitation, acupuncture, use of bracing, among others) in the last 2-months; corticosteroid IAI in the joint understudy in the last 3-months; suspected local or systemic infection; suspected pregnancy; simple radiography of the hands suggesting IP arthropathy of another etiology (psoriatic arthritis, microcrystalline arthropathy, deposition disease); and severe coagulation disorder.

### Intervention

Patients were randomized into two groups: Study group: TH/LD; Control group: LD.

Patients in the TH/LD group underwent one IAI at their most symptomatic IP (single joint) with TH (20 mg/mL) and LD 2% without a vasoconstrictor. TH 0.3 mL (6 mg) was used for the PIPs and TH 0.2 mL (4 mg) for the DIPs. In this group, TH was always administered with 0.1 mL of 2% LD.

Patients in the LD group were submitted to one IAI with 0.1 mL of 2% LD without a vasoconstrictor (at their most symptomatic IP, single joint).

For both groups, oral use of acetaminophen, 750 mg/tablet, as required (up to three tablets per day). The IAI in both groups was administered by the same rheumatologist with 20-years of experience in interventional rheumatology; the injection was administered following rigorous antisepsis with 0.5% alcoholic chlorhexidine. Sterile BD Ultra-Fine^TM^ syringes with 8 mm  ×  0.3 mm (30G) needles covered with opaque adhesives were used on all patients. The anatomical repair used for needle entry was the point located at the junction between the dorsal fold of the joint and the line formed by the meeting of the dorsal and lateral finger faces.^11^ After the procedure, the injection joint was immobilized with a splint for 48 hours.

Patients in both groups were instructed not to introduce or discontinue any new drug or non-drug intervention for the treatment of OA during the entire follow-up of the study.

### Outcomes

The patients had their data recorded on an evaluation form. The most important aspects of the study were age, gender, skin color, and the use of chondroprotective drugs, anti-inflammatories, and/or analgesics.

Six clinical assessments were scheduled for a total of 48-weeks of follow-up: T0 (before the intervention), T1 (1-week after the intervention), T4 (4-weeks), T8 (8-weeks), T12 (12-weeks), and T48 (48-weeks). Blinded assessors, trained to administer the assessment instruments, carried out the assessments.

Patients were followed up at assessment times through appointments scheduled by the study's principal investigator (1-NOSP) and were assessed "blindly" by two observers, a physical therapist (4-HAVO) and a rheumatologist (3-FSM).

### Clinical assessment

The following variables were assessed in both groups:

• Visual Analogical Score (VAS) of pain at rest (VASr of 0–10 cm, self-reported);
• VAS for pain on movement (VASm of 0–10 cm, self-reported);
• VAS for joint swelling (physician VASs of 0–10 cm, physician assessed).

### Joint goniometry in flexion (degrees of range motion);

• Analgesics consumption (daily mean of acetaminophen)*;
• Grip strength using the Jamar dynamometer (kgf) by obtaining the average of three attempts;^12^
• Pinch strength using the Pinch Gauge dynamometer (kgf) (the average of three attempts for the three types of pinches: pulp–pulp, key, and tripod;^12^
• Hand function was assessed by the Brazilian version of the COCHIN^13^ questionnaires and the AUSCAN OA index, which evaluated pain, stiffness, and hand function;^14^
• An evaluation scale that assessed improvement across 5 points (much worse, worse, unchanged, little improvement, and much improvement);^15^
• Adverse effects after the procedure (atrophy and/or subcutaneous atrophy and joint instability); and
• Worsening of pain after IAI, as measured by VAS (post IAI VAS 1–10 cm) 48 hours after the procedure (reposted only at T1).

*Acetaminophen consumption was assessed by filling in a daily calendar with all study days.

### Radiographic assessment

Radiographic assessments were performed by a blinded musculoskeletal radiologist who had 30-years of experience. Conventional radiography was performed on the hands at T0 and T48.

The articular radiographic images were classified according to the Kellgren and Lawrence score;^16^ they were also examined for the presence or absence of erosive OA^17^ and for any worsening between the two images.

### Sample size

Using the VASr as the primary study variable, the authors identified that a sample of 24 patients for each group would ensure adequate power. To arrive at the studied sample, the authors considered a Standard Deviation (SD) equal to 1.5 points based on previous studies. The authors also used analysis of variance (ANOVA) for repeated measures as the statistical method to calculate the sample. The statistical power was 90%, with 5% significance, and with a detectable difference of 2.0 points on the VAS pain scale when compared with the control group, measured six times across two independent groups. Anticipating a possible loss, the authors started with 30 patients in each group.

### Random selection

Patients were randomly assigned using a randomization schedule generated using the MINITAB 14.0 software without any stratification factors; secret allocation was guaranteed by opaque, sealed envelopes. The rheumatologist responsible for the inclusion of these patients had no previous access to the randomization list. That rheumatologist was responsible for making sure the patients were within the inclusion and exclusion criteria of the present study and, after the procedure, for referring patients to the evaluators in another room.

### Sample blinding

Only the researcher responsible for the patients' inclusion and exclusion had access to which group the patients belonged after enrollment. The observer responsible for patient assessments was completely blinded to the present study. The rheumatologist performing the procedure had no access to the recruitment, random allocation, inclusion criteria, and patient assessments; otherwise, study blinding may have been impaired due to the fact that the volume of those in the TH/lidocaine (study) group was greater than that of the LD (control) group.

### Statistical analysis

SPSS software version 17.0 (IBM Corporation, Armonk, NY, USA) was used to perform the statistical analysis of the data. Descriptive statistics (mean, SD, 95% Confidence Intervals [CIs]) were used to characterize the patients in each group. Continuous variables of the two groups at baseline were compared using Student's *t*-test for normally distributed variables and the Mann-Whitney test for variables with a distribution not considered normal. Categorical variables were assessed using Pearson's Chi-Squared test.

To evaluate the response to the intervention, ANOVA was used for repeated measures for the intergroup and intragroup analyses over time. Differences were considered statistically significant when p < 0.05.

## Results

**Fig. 1:**
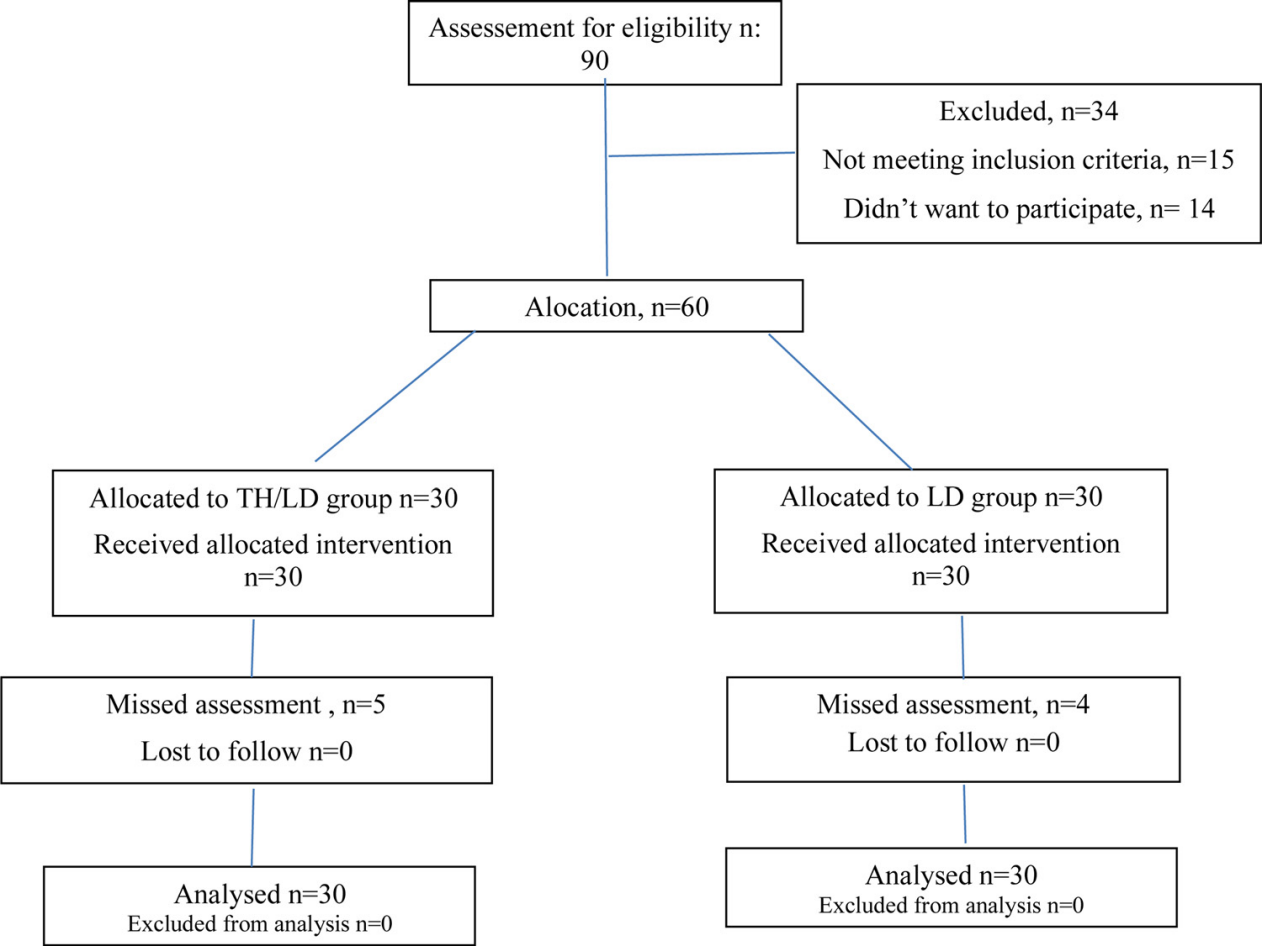
Study Flow Diagram. TH/LD, Triamcinolone Hexacetonide group; LD, Lidocaine group.

The study design is described in flow diagram 1 (Fig. 1).^18^ Sixty patients were studied overall; 97% were women, and the patients had a mean age of 61-years (SD = 8.2) and had hand OA for approximately 5-years (3.6). No differences were found between groups in terms of age, disease duration, gender, and other baseline variables (Table 1). The groups differed only in relation to skin color. There were no patients in the present study's sample using non-drug treatment at baseline, and there were no patients who started this type of treatment during the entire study follow-up in either group. All patients reached the end of the study. There were nine missed assessments in total – 5 in the TH/LD group and 4 in the LD group. These absences in the assessment times were due to difficulty in attending appointments. All assessments were available for T1 and T48.

**Table 1:** Sample characteristic at baseline.

	TH/LD Group (n = 30)	LD Group (n = 30)	p
Age – years			
Mean (SD)	60.7 (9.1)	60.7 (7.3)	0.553^a^
Disease time onset ‒ years			
Mean (SD)	4.7 (4.2)	5.2 (3.0)	0.151^a^
Gender			
Female/Male, n (%)	30 (100%)/0	28 (93.3%)/2 (6.7%)	0.15^b^
Skin color			
White/ no white, n (%)	17 (56.7%)/13 (43.3%)	25 (83.3%)/5 (16.7%)	0.034^b^
Kelgreen Lawrence (injected joint)			0.180^b^
I	8 (27%)	6 (20%)	
II	9 (30%)	7 (23%)	
III	7 (23%)	9 (30%)	
IV	6 (20%)	8 (27%)	
X-Ray erosion			0.297^b^
No erosion	19 (63.3%)	15 (50%)	
Erosion	11 (36.7%)	15 (50%)	
Drugs			0.423^b^
No drugs	23 (76.7%)	18 (60%)	
Hydroxychloroquine	1 (3.3%)	2 (6.7%)	
Glucosamine sulfate	5 (16.7%)	5 (16.7%)	
Glucosamine sulfate + chondroitin sulfate	0	3 (10%)	
Methotrexate	1 (3.3%)	1 (3.3%)	
Interphalangeal joint studied			
DIP, n (%)	14 (46.7%)	15 (50%)	0.796^b^
PIP, n (%)	16 (53.3%)	15 (50%)	0.423^b^

TH/LD, Triamcinolone Hexacetonide/Lidocaine; LD, Lidocaine; SD, Standard Deviation; KL, Kellgren-Lawrence classification scale; DIP, Distal Interphalangeal joint; PIP, Proximal Interphalangeal joint.

a Mann-Whitney statistical test.

b Chi-Square statistical test.

### Analysis of joint pain, swelling, and flexion

The statistical comparison between groups for joint pain at rest, movement, joint swelling, and joint flexion can be seen in Table 2. For VASr, the TH/LD and LD groups started from an average of 6.1 and 6.1 at T0 to 0.3 and 0.5 at T48, respectively; for the VASm, the groups started from an average of 6.5 and 6.6 at T0 to 2.6 and 3.0 at T48, respectively; for joint flexion, the groups started from an average of 71.6 and 61.1 at T0 to 66.7 and 62.2 at T48, respectively. For all these variables, there was no statistical difference between the groups. Patients in the TH/LD group had a better evolution in VAS swelling over time at T48 (p = 0.04). This group had an average of VAS swelling of 3.0 at T0, evolving to 0.8 at T48, while the LD group had an average of 3.0 at T0 and evolved to 1.2 at T48. The TH/LD group showed lower VAS swelling averages than the LD group at all times after T0. For all repeated continuous variables in Table 2, there were a statistical intragroup difference in relation to T0. For the VAS 48h after injection, there was no statistical difference between the groups (Table 2).

**Table 2:** Comparison between groups for pain, swollen, joint goniometry and pain 48 hours after procedure.

Time (weeks)	TH/LD Group (n = 30)	LD Group (n = 30)	p
VASr – Mean (SD)			0.599^a^
T0	6.1 (1.7)	6.1 (1.6)	
T1	2.6 (2.9)	1.7 (2.7)	
T4	1.3 (2.1)	1.6 (2.6)	
T8	1.4 (2.6)	1.6 (2.6)	
T12	0.8 (1.7)	0.9 (2.2)	
T48	0.3 (1.3)	0.5 (1.5)	
p intragroup	<0,001	<0,001	
VASm – Mean (SD)			0.063^a^
T0	6.5 (1.8)	6.6 (1.4)	
T1	3.9 (3.1)	4.1 (2.9)	
T4	2.8 (2.9)	3.0 (3.0)	
T8	1.8 (2.6)	4.0 (3.3)	
T12	2.2 (2.9)	4.0 (3.2)	
T48	2.6 (3.3)	3.0 (3.0)	
p intragroup	< 0,001	< 0,001	
VASs – Mean (SD)			0.040^a^
T0	3.0 (1.5)	3.0 (1.7)	
T1	2.0 (1.5)	2.1 (1.4)	
T4	1.4 (1.4)	2.0 (1.2)	
T8	0.7 (0.8)	1.8 (1.3)	
T12	1.1 (1.2)	2.0 (1.3)	
T48	0.8 (0.9)	1.2 (1.1)	
p intragroup	< 0,001	< 0.001	
VAS 48h after injection ‒ Mean (SD)	3.56 (3.2)	3.8 (3.4)	0.825^b^
Joint Flexion (^o^) – Mean (SD)			0.659^b^
T0	71.6 (20.4)	61.1 (19.8)	
T1	73.4 (22.8)	66.6 (20.6)	
T4	75.6 (22.3)	68.7 (19.1)	
T8	79.3 (21.1)	66.2 (19.4)	
T12	72.6 (25.1)	63.6 (22.6)	
T48	66.7 (26.5)	62.2 (23.4)	
p intragrupo	0.012	0.012	

TH/LD, Triamcinolone Hexacetonide/Lidocaíne; LD, Lidocaíne; SD, Standard Deviation; VASr, Visual Analogic Scale for rest pain; VASm, Visual Analogic Scale for movement pain; VASs, Visual Analogic Scale for joint swollen.

Statistical test:

a ANOVA for repeated measures;

b Test *t* de Student

### Analysis of pinch and grip strength

There was no statistical difference between the groups for the variables Grip strength, key pinch, pulp-to-pulp, and tripod pinch (Table 3). Statistical improvement in the intragroup evaluation was observed for both groups, for the strength of the pulp-to-pulp and tripod pinch (using the finger of the joint studied) (p < 0.001). However, no intragroup statistical difference was observed for Grip strength (p = 0.093) and key pinch (p = 0.066) (Table 3).

**Table 3:** Comparison between groups for grip and pinch strength.

Time (weeks)	TH/LD Group (n = 30)	LD Group (n = 30)	p
Grip strength (Kgf) – Mean (SD)			0.976
T0	14.8 (6.7)	13.7 (7.6)	
T1	14.1 (6.6)	13.7 (7.8)	
T4	15.1 (6.6)	14.6 (7.8)	
T8	15.5 (7.3)	15.4 (7.4)	
T12	16.2 (6.2)	15.2 (7.7)	
T48	17.0 (7.0)	16.7 (6.7)	
p intragroup	0.093	0.093	
Key pinch strength (kgf) – Mean (SD)			0.679
T0	6.1 (1.8)	5.9 (2.4)	
T1	5.9 (1.8)	6.3 (1.9)	
T4	6.4 (1.6)	6.3 (2.1)	
T8	6.4 (2.0)	6.5 (1.6)	
T12	6.5 (1.9)	6.2 (1.7)	
T48	6,7 (1.9)	6.6 (1.8)	
p intragroup	0.066	0.066	
Tip pinch strength (kgf) – Mean (SD)			0.641
T0	2.8 (1.2)	2.6 (1.3)	
T1	2.8 (1.2)	3.0 (1.4)	
T4	3.3 (1.1)	3.0 (1.4)	
T8	3.3 (1.3)	3.3 (1.6)	
T12	3.4 (1.2)	3.4 (1.7)	
T48	3.5 (1.5)	3.7 (1.6)	
p intragroup	<0.001	<0.001	
Tripod pinch strength (kgf) – Mean (SD)			0.232
T0	4.1 (1.8)	3.8 (2.0)	
T1	4.1 (1.6)	4.1 (2.0)	
T4	4.5 (1.6)	4.2 (1.9)	
T8	4.8 (1.8)	4.5 (2.0)	
T12	4.8 (1.9)	4.5 (2.0)	
T48	4.5 (1.8)	5.0 (2.0)	
p intragroup	<0.001	<0.001	

TH/LD,Triamcinolone Hexacetonide/Lidocaine; LD, Lidocaine; SD, Standard Deviation; Kgf, Kgforce; Statistical test: ANOVA for repeated measures.

### Analysis of the COCHIN functional questionnaire and AUSCAN Index

The two groups behaved similarly for the COCHIN functional questionnaire and AUSCAN Index, with no intergroup statistical difference (Table 4). Improvements across the AUSCAN global, AUSCAN pain, and AUSCAN function scores were observed in the intragroup assessment for both groups. However, there was no statistical significance in the intragroup analysis for the COCHIN functional questionnaire (p = 0.07) e AUSCAN-stiffness (p = 0.088) (Table 4).

**Table 4:** Comparison between groups regarding functional questionnaire.

Time (weeks)	TH/LD Group (n = 30)	LD Group (n = 30)	p
COCHIN – Mean (SD)			0.709
T0	19.3 (17.3)	23.1 (16.3)	
T1	17.8 (19.5)	22.1 (16.7)	
T4	14.3 (16.4)	19.9 (16.7)	
T8	15.9 (18.1)	20.5 (16.2)	
T12	14.3 (15.2)	21.8 (16.7)	
T48	14.3 (14.7)	16.2 (13.6)	
p intragroup	0.07	0.07	
AUSCAN-global – Mean (SD)			0.626
T0	25.9 (15.1)	29.1 (13.4)	
T1	22.7 (13.8)	27.5 (15.2)	
T4	20.0 (13.9)	25.5 (14.0)	
T8	20.3 (14.6)	26.0 (14.0)	
T12	18.8 (14.1)	25.7 (14.4)	
T48	18.9 (11.4)	20.9 (14.1)	
p intragroup	<0.001	<0.001	
AUSCAN-pain – Mean (SD)			0.593
T0	8.8 (4.7)	9.2 (4.3)	
T1	7.0 (4.6)	8.3 (5.1)	
T4	5.9 (4.9)	7.8 (5.4)	
T8	6.0 (4.9)	8.3 (4.5)	
T12	5.3 (4.7)	7.0 (4.8)	
T48	5.4 (4.0)	6.3 (3.9)	
p intragroup	<0.001	<0.001	
AUSCAN-stiffness – Mean (SD)			0.698
T0	1.7 (1.4)	2.0 (1.5)	
T1	1.1 (1.3)	1.5 (1.5)	
T4	1.2 (1.3)	1.8 (1.5)	
T8	1.6 (1.4)	1.9 (1.2)	
T12	1.2 (1.4)	2.0 (1.4)	
T48	1.4 (1.3)	1.8 (1.5)	
p intragroup	0.088	0.088	
AUSCAN-function – Mean (SD)			0.886
T0	15.4(10.4)	17.9 (8.9)	
T1	14.6 (9.5)	17.8 (9.7)	
T4	12.9 (8.9)	15.9 (8.8)	
T8	12.7 (9.5)	15.8 (9.8)	
T12	12.3 (9.8)	16.7 (9.8)	
T48	12.1 (8.2)	14.0 (9.1)	
p intragroup	0.005	0.005	

TH/LD, Triamcinolona Hexacetonide/lidocaine; LD, Lidocaine; SD, Standard Deviation; Statistical test: ANOVA for repeated mean.

### Analysis of acetaminophen consumption and perception of improvement

For the daily consumption of acetaminophen, a statistically significant reduction was observed in the intragroup analysis, but only for the TH/LD group (TH/LD p = 0.019 vs. LD p = 0.488) (Table 5). In the intergroup analysis for this variable, it was observed that patients in the TH/LD group demonstrated improvements over time when compared with the LD group (p < 0.001). Further, there was an increase in acetaminophen consumption in both groups in the first evaluation period following the IAI (T1). However, in the TH/LD group, the consumption fell at T48, while in the LD group, consumption increased in that time frame. Conversely, regarding the perception of improvement, as based on the improvement scale (worse, unchanged, and improve), patients demonstrated the same trends in both groups, with no statistical difference between groups (p = 0.236). It is observed that the perception of improvement remained stable in both groups from T1 to T48, therefore also without an intragroup statistical difference (p = 0.329 for both groups) (Table 5).

**Table 5:** Comparison between groups regarding acetaminophen use, scale of subjective improvement and radiographic assessment.

Tempos (semanas)							TH/LD Group (n = 30)		LD Group (n = 30)		p intergroup
Acetaminophen daily consuption – Mean (unit-750 mg) (SD)											<0.001^a^
T0							0.65 (1.12)		0.17 (0.45)		
T1							0.89 (1.21)		0.31 (0.58)		
T4							0.71 (1.15)		0.26 (0.49)		
T8							0.81 (1.19)		0.30 (0.58)		
T12							0.74 (1.25)		0.33 (0.60)		
T48							0.14 (0.43)		0.38 (0.76)		
p intragrupo							0.019		0.488		
Improvement scale, n (%)											0.236^b^

	TH/LD Group						LD Group				
	Worse		Unchanged		Improve		Worse		Unchanged		Improve

T1	2 (6.9%)		1 (3.4%)		27 (90%)		0		1 (3.3%)		29 (96.7%)
T4	0		1 (3.3%)		29 (96.7%)		0		4 (13.3%)		26 (86.7%)
T8	1 (3.3%)		1 (3.3%)		28 (93.3%)		1 (3.3%)		3 (10%)		26 (86.7%)
T12	2 (6.7%)		1 (3.3%)		27 (90%)		1 (3.3%)		5 (16.7%)		24 (80%)
T48	1 (3.3%)		5 (16.7%)		24 (80%)		5 (16.7%)		2 (6.7%)		23 (76.6%)
p intragroup	0.329						0.329				

Radiographic assessment in the 48-weeks follow-up											p
		TH/LD Group (n = 30)					LD Group (n = 30)				0.564^a^
KL Classification		1	2	3		4	1	2	3	4	

T0		8 (27%)	9 (30%)	7 (23%)		6 (20%)	6 (20%)	7 (23%)	9 (30%)	8 (27%)	
T48		5 (17%)	7 (23%)	12 (40%)		6 (20%)	3 (10%)	7 (23%)	12 (40%)	8 (27%)	
p intragroup		< 0.001					< 0.001				

Erosion											p
		TH/LD Group (n = 30)					LD Group (n = 30)				0.999^a^
OA classification (erosion)		No erosion		Erosion			No erosion		Erosion		

T0		19 (63%)		11 (37%)			15 (50%)		15 (50%)		
T48		18 (60%)		12 (40%)			14 (47%)		16 (53%)		
p intragroup		0.157					0.157				

Radiographic evolution –worse/ unchanged at 48-weeks											p
		TH/LD Group (n = 30)					LD Group (n = 30)				0.573^c^

Unchanged		20 (67%)					22 (73%)				
Worse		10 (33%)					8 (27%)				

TH/LD, Triamcinolone Hexacetonide/Lidocaíne; LD, Lidocaíne; SD, Standard Deviation; KL, Kellgren e Lawrence scale; OA, Osteoarthritis.

Statistical test: ^a^ ANOVA for repeated measures

b ANOVA for repeated measures for categorical variables

c Qui-Square test.

### Radiographic assessment

As can be seen in Table 5, both groups presented worsening (intragroup assessment) according to KL classification, mainly in relation to classification from KL2 to KL3 (p < 0.001). From T0 to T48, the number of patients with KL3 increased from 7 to 12 and from 9 to 12, respectively, in the TH/LD and LD groups. No statistical difference was observed in the intergroup analysis for the radiographic classification of KL (p = 0.564). There were no statistical differences in the presence of erosions over 48-weeks in both groups (intragroup p = 0.157). From T0 to T48, the number of patients with erosion increased from 11 to 12 and from 15 to 16, respectively, in the TH/LD and LD groups, also with no difference in the intergroup analysis (p = 0.999).

### Side effects

No significant side effects were observed in the present study. Patients reported experiencing mild discomfort associated with IAI in both groups. The mean worsening of joint pain following IAI was very similar in both groups, as shown in Table 2. In only one patient was observed deformity (maintained flexion) in the Interphalangeal Distal (IFD) joint in the finger that suffered IA (with TH/LD injected in the Interphalangeal Proximal [IFP] joint 12-weeks after the procedure).

## Discussion

The authors aimed to evaluate the effectiveness of IAI with TH for hand OA and the associated effects of this intervention on patients' radiographic changes over time. The authors observed that an IAI of TH was effective at improving joint swelling and reducing the daily consumption of analgesics over time; however, the authors did not observe any influences of this procedure on the radiographic evolution of the injected joint.

The midterm results (12-weeks) with the same sample of patients had already been published, demonstrating superiority in the TH/LD group for joint swelling and pain on movement.^10^ This publication on IAI was considered a well-designed study with low risk of bias in a 2016 systematic review about the use of an IAI on hand OA.^1^ The strengths of this first study are due to the fact that it was a prospective, randomized, controlled, double-blind study with a 12-week follow-up. This study[10] is among the consulted literature in the most recent European League Against Rheumatism (EULAR) recommendations for the treatment of the hands OA.^19^ This task force recognized that, in specific cases where joint inflammation is present, IAI with a corticosteroid might be a therapeutic option.^19^ In order to evaluate the long-term effectiveness of this procedure and its effects on radiographic changes, the authors chose to follow these patients for 48-weeks in the present study.

When examining other evaluations of the effectiveness of intra-articular corticosteroids in the treatment of OA in the literature, its use is recommended, according to some reviews.^20^ There are several drugs that can be used in IAI, such as hyaluronic acid, platelet-rich plasma, hypertonic dextrose, ozone, and regenerative therapy.^21^ The Italian society of Rheumatology recommends the use of IAI corticosteroids as the first treatment option for asymptomatic first carpometacarpal,^22^ although the great majority of the studies articulated doubts about indications for the procedure, primarily associated with the lack of evidence of its effectiveness. When reviewing the literature, the authors found several randomized controlled trials that used IAI for the treatment of hand OA. However, these studies mainly involved the first carpometacarpal joint. These studies had conflicting results.^23^, ^24^, ^25^, ^26^, ^27^, ^28^ The difficulty in comparing these studies is due to the different points studied: type of joint injected, type of corticosteroid used, absence of a control group, or a blinded assessment.

Few studies have analyzed the effectiveness of the IAI of corticosteroids exclusively in the IP in hand OA. In the study of Reeves et al.^23^ thirteen interphalangeal OA patients received intra-articular dextrose and xylocaine, and fourteen patients received only xylocaine at 0, 2 , and 4-months. The patients were assessed at 6-months according to rest, movement and grip VAS pain, and joint flexion range of motion and at 12-months according to radiographic joint narrowing grade, osteophyte grade, and joint width (mm). VAS of pain on movement (42% vs. 15%; p = 0.027) and flexion range of motion (+8 degrees vs. -8.6 degrees; p = 5.003) were better in the dextrose group. The joint narrowing grade improved more in the dextrose-treated patients (p = 0.006). Miller et al.^29^ conducted a prospective "uncontrolled" study with fifty patients who received interphalangeal intra-articular steroid-guided injections. These patients were assessed at six weeks, three, and six months according to the duration of pain relief, hand function, and range of movement. There were significant improvements in pain scores, range of movement, and hand function for up to three months in these patients.

In agreement with the present study, only the studies of Fuchs et al.^25^ and Meenagh et al.^27^ performed IAI with TH; however, these authors administered it in the carpometacarpal joints and demonstrated opposing results between them.

In the present study, the authors used several assessment tools that helped to validate the results. The authors chose to measure variables of pain, swelling, goniometry, hand function, hand strength, and finger strength. Intragroup improvement was observed for the vast majority of outcomes assessed in both groups in the present study.

When examining hand and pinch strength, only pulp-to-pulp and tripod pinch had intragroup improvements.

In this study, the authors used the corticosteroid TH. Among the various IAI corticosteroids used in OA, TH appears to be a more effective option. In a comparative study with betamethasone, Valtonen et al.^30^ achieved better and longer-lasting results with TH.

The TH dose used in the present study was chosen empirically. In the studies by Furtado et al.^31^ and Lopes et al.,^32^ 0.5–1 mL of TH was used in the metacarpophalangeal joints. The authors then chose to use a smaller dose in the IP studied. Because the use of IAI of IPs is a potentially painful procedure, it was decided to use lidocaine (0.1 mL), not saline, in both groups in the procedure.

In spite of the intragroup improvements observed across almost all variables examined in the present study, the authors obtained a statistically significant intergroup difference at 48-weeks of follow-up for joint swelling and acetaminophen consumption, with superiority observed for the TH/LD group. That is, joint swelling demonstrated improvements at 12-weeks, and this was maintained over the course of 1-year. Indirectly consuming fewer analgesics suggests clinical improvements among patients injected with TH. It should be noted that the joint evaluated in the present study was injected only once (T0), and even so, joint swelling still demonstrated an improvement after 48-weeks.

Although the authors observed superiority for the TH/LD group for very important variables, attention was drawn to the similarity in the changes observed between the two groups across the majority of variables studied. This may be related to several factors. Based on the baseline VAS, VASm, and VASs scores, the present study's sample of patients had reported more pain than joint swelling. With a larger sample, a difference could be found between the groups for a greater number of variables. The small effect on grip or key pinch strength and, even less, on the overall assessment of the patient can be due to the only one finger treatment (the most symptomatic).

The dose of TH used may have been small; however, there are no studies determining the optimal dose of TH for the IP joints. The 36-week distance between T12 and T48 may have influenced the decrease in the effect of IAI on joint pain and the similarity between the groups at T48. Finally, perhaps the use of lidocaine as a controlled drug had a greater and longer-lasting analgesic effect, more than expected, even in the LD group. The authors believe that this last factor was important for explaining the similar changes observed between the groups in the present study. If the authors had used saline solution or just the introduction and exit of the needle in the control group, the present study's results may have demonstrated greater differences between groups.

The effects of lidocaine on cartilage have been widely questioned. In a review of its deleterious effects, Piper et al.^33^ in 2011 cautioned against the use of IAI anesthetics in high concentrations, although they have not focused on the use of single doses. Some in vitro studies have warned of the deleterious effect of lidocaine on cartilage,^34^, ^35^, ^36^, ^37^, ^38^, ^39^ and even in a single dose.^40^ On the other hand, in 2012, Piat et al.^41^ suggested that there was an anabolic effect on cartilage metabolism based on increases in cartilaginous synthesis markers following the administration of anesthetics. On the contrary, there is a possible anti-inflammatory effect attributed to this anesthetic, according to some authors. Olsen et al.^42^ demonstrated an anti-inflammatory effect of an inhaled lidocaine analogue, and this subject had also been discussed when speaking of other local anesthetics.^43^ In the present study, the anti-inflammatory effect of lidocaine, as well as its inhalational use, may have been responsible for the similarity of the IAI effect between the two groups.

The authors found good tolerance to the procedure in the two groups studied, with no difference between them in terms of discomfort and the worsening of pain immediately after the procedure; further, significant side effects were not observed. This suggests that IP IAIs represent a viable clinical procedure when performed by a trained rheumatologist.

There are controversies associated with the safety of "repeated" corticosteroid IAIs. Two well-designed studies on this subject have been conducted on knee IAI. First, in 2003, some authors observed that in a controlled study of IAI corticosteroids versus saline, an absence of radiographic worsening in the IAI group was evident every 3-months over the course of 2-years with the injection of acetonide and triamcinolone.^44^ Second, very recently, another controlled study observed a greater loss of volume of articular cartilage, as observed during magnetic resonance imaging; further, there was also an absence of improvements in pain following the use of corticosteroid IAIs administered every 3-months over the course of a 2-year follow-up.^45^

In the present study, the authors were interested in exploring the possible deleterious effects of TH IAI on the radiographic evolution of the injected joint; the authors thus chose to follow these joints radiologically for 48-weeks. The injected joint of both groups behaved very similarly throughout the 48-weeks of the study, based on: KL classification, the presence of bone erosions, and the OA worsening. The findings suggested that the use of a single IAI with a corticosteroid, which is considered to be more potent in a very small joint with OA, was not associated with radiographic changes. Despite similarities between the groups in terms of radiological evolution, radiographic worsening was observed in both groups according to the KL radiographic classification grade 2 to grade 3. This worsening may be due to the natural course of the disease. However, it is possible that lidocaine toxicity on the articular cartilage influenced this radiographic worsening in both groups.

The present study has limitations. First, the choice to examine a single joint in response to an intervention and the use of lidocaine as a control group are the main factors that may have compromised the present study's results. The difference in the volume injected in the two groups may have also impaired the results. An analysis of the effect size was not obtained. Adding another group with a saline injection to the protocol would respond better to the hypothesis of radiographic progression (considering the potential deleterious effect of lidocaine on cartilage). Also, this other group would add more power to the study, as the lidocaine injection could not be inert and may have diluted the positive outcomes in the intervention group. Finally, the 9-month gap between the assessments at T12 and T48 may have compromised the detection of improvements in the TH/LD group within that time interval.

Overall, the present study shows that a simple and inexpensive procedure, such as the use of IAI with TH, can be a safe and effective option for improving joint swelling and reducing the use of analgesics in patients with hand OA, and this is not associated with radiographic changes.

Further studies are needed to evaluate the effectiveness and safety of this procedure in the long term and with a greater number of patients.

## Conclusion

The IAI of TH in the IP joint of patients with hand OA is effective for improving joint swelling while reducing analgesic consumption in the long term (48-weeks). Further, the use of IAI TH does not appear to be associated with radiographic changes in these patients.

## Funding

Clinical Trials.gov (NCT02102620).

Local ethics committee subscription: Unifesp ‒ CEP 0956/11.

## Ethical approval and consent to participate

Approved by the local ethics committee (UNIFESP ‒ CEP 0956/11) and all recruited patients provided their written and informed consent.

## Consent for Publication

All authors authorized publication.

## Availability of Data and Materials

Patients' evaluation sheets are with the corresponding author.

## Authors' contributions

Paschoal NOS: Principal author, rheumatologist responsible for recruiting patients and organizing the protocol. Natour J: Rheumatologist, study's co-advisor, and author of the article. Machado FS: Rheumatologist responsible for clinical examination. Oliveira HAV: Physiotherapist responsible for clinical examination. Fernandes ARC: Radiologist responsible for X-Ray examination. Furtado RNV: Rheumatologist, study's advisor and author of the article.

## Conflicts of interest

The authors declare no conflicts of interest.

## References

[1] Kroon FPB, Rubio R, Schoones JW. Intra-articular therapies in the treatment of hand osteoarthritis: a systematic literature review. Drugs Aging. 2016;33:119-33. 10.1007/s40266-015-0330-5 PMC4756050 26650235

[2] Kloppenburg M, Boyesen P, Smeets W, Haugen I, Liu R, Visser W (2014). Report from the OMERACT hand osteoarthritis special interest group: advances and future research priorities. J Rheumatol..

[3] Roemer FW, Eckstein F, Hayashi D, Guermazi A. (2014). The role of imaging in osteoarthritis. Best Pract Res Clin Rheumatol..

[4] Mahendira D, Towheed TE. (2009). Systematic review of non-surgical therapies for osteoarthritis of the hand: an update. Osteoarthr Cartilage..

[5] Dougados M., Hochberg MC, Silman AJ, Smolen JS, Weinblatt ME, Weisman MH (2007). Rheumatology.

[6] Hochberg MC, Altman RD, April KT. (2012). American college of rheumatology 2012 recommendations for the use of nonpharmacologic and pharmacologic therapies in osteoarthritis of the Hand, Hip, and Knee. Arthrit Care Res..

[7] Bruyere O, Cooper C, Pelletier JP, Maheu E, Rannou F, Branco J (2016). A consensus statement on the European Society for Clinical and Economic Aspects of Osteoporosis and Osteoarthritis (ESCEO) algorithm for the management of knee osteoarthritis – From evidence-based medicine to the real-life setting. Semin Arthritis Rheum..

[8] McAlindon TE, Bannuru RR, Sullivan MC, Arden NK, Berenbaum F, Bierma-Zeinstra SM (2014). OARSI guidelines for the non-surgical management of knee osteoarthritis. Osteoarthr Cartilage..

[9] Bellamy N, Campbell J, Welch V, Gee TL, Bourne R, Wells GA. (2006). Intraarticular corticosteroid for treatment of osteoarthritis of the knee. Cochrane Database Syst Ver..

[10] Spolidoro-Paschoal NO, Natour J, Machado FS, Oliveira HAV, Furtado RNV (2015). Effectiveness of triamcinolone hexacetonide intraarticular injection in interphalangeal joints: a 12-week randomized controlled trial in patients with hand osteoarthritis. J Rheumatol..

[11] Furtado R, Natour J. (2011). Infiltrações apendiculares de membro superior, em: Infiltrações do Aparelho Locomotor.

[12] Mathiowetz V, Weber K, Volland G, Kashman N. (1984). Reliability and validity of grip and pinch strength evaluations. J Hand Surg..

[13] Chiari A, Souza CC, Natour J. (2011). Translation, cultural adaptation and reproducibility of the Cochin Hand Functional Scale questionnaire for Brazil. Clinics.

[14] Freitas PJP, Dias RCD. (2010).

[15] Likert Rensis. (1932). A technique for the measurement of attitudes. Arch Psychol..

[16] Kellgren JH, Lawrence JS. (1957). Radiological assessment of osteoarthrosis. Ann Rheum Disease..

[17] Peter JB, Pearson CM, Marmon L. (1966). Erosive osteoarthritis of the hands. Arthritis Rheum..

[18] Schulz KF, Altman DG, Moher D, for the CONSORT Group (2010). CONSORT 2010 statement: updated guidelines for reporting parallel group randomized trials. BMJ.

[19] Kloppenburg M, Kroon FPB, Blanco FJ, Doherty M, Dziedzic KS, Greibrokk E (2019). 2018 update of the EULAR recommendations for the management of hand osteoarthritis. Ann Rheum Dis..

[20] Ayral X. (2001). Injections in the treatment of osteoarthritis. Best Pract Res Clin Rheumatol..

[21] Testa G, Giardina SMC, Culmone A, Vescio A, Turchetta M, Canavo S (2021). Intra-articular injections in knee osteoarthritis: a review of literature. J Funct Morphol Kinesiol..

[22] Manara M, Bortoluzzi A, Favero M, Prevete I, Scire C, Bagnato G (2013). Italian Society for Rheumatology recommendations for the management of hand osteoarthritis. Reumatismo..

[23] Reeves KD, Hassanein K. (2000). Randomized, prospective, placebo-controlled double-blind study of dextrose prolotherapy for osteoarthritic thumb and finger (DIP, PIP, and trapeziometacarpal) joints: evidence of clinical efficacy. J Altern Complement Med..

[24] Figen Ayhan F, Ustun N. (2009). The evaluation of efficacy and tolerability of Hylan G-F 20 in bilateral thumb base osteoarthritis: 6 months follow-up. Clin Rheumatol..

[25] Fuchs S, Monikes R, Wohlmeiner A, Heyse T. (2006). Intra-articular acid compared with corticoid injections for the treatment of rhizarthrosis. Osteoarthr Cartilage..

[26] Roux C, Fontas E, Breuil V, Brocq O, Albert C, Euller-Ziegler L. (2007). Injection of intra-articular sodium hyaluronidate (sinovial) into the carpometacarpal joint of the thumb in osteoarthritis. A prospective evaluation of efficacy. Joint Bone Spine.

[27] Meenagh GK, Patton J, Kynes C, Wright GD. (2004). A randomized controlled trial of intra-articular corticosteroid injection of the carpometacarpal joint of the thumb in osteoarthritis. Ann Rheum Dis..

[28] Joshi R. (2005). Intraarticular corticosteroid injection for first carpometacarpal osteoarthritis. J Rheumatol..

[29] Miller CA, Dalgleish S, Cox Q. (2017). X-ray guided steroid injections for proximal interphalangeal joint osteoarthritis of the fingers. J Hand Surg Am..

[30] Valtonen EJ. (1981). Clinical comparison of triancinolonehexacetonide and betamethasone in the treatment of osteoarthrosis of the Knee-joint. Scand J Rheumatol..

[31] Furtado RNV, Oliveira LM, Natour J. (2005). Polyarticular corticosteroid injection versussystemic administration in treatment of rheumatoid arthritis patients: a randomized controlled study. J Rheumatol..

[32] Lopes RV, Furtado RNV, Parmigiani L, Rosenfeld A, Fernandes ARC, Natour J. (2008). Accuracy of intra-articular injections in peripheral joints performed blindly in patients with rheumatoid arthritis. Rheumatology..

[33] Piper SL, Kramer JD, Kim HT, Feeley BT (2011). Effects of local anesthetics on articular cartilage. Am J Sports Med..

[34] Lo IKY, Sciore P, Chung M, Liang S, Boorman RB, Thornton GM (2009). Local anesthetics induce chondrocyte death in bovine articular cartilage disk in a dose – and duration-dependent manner. Arthroscopy..

[35] Jacobs TF, Vansintjan PS, Roels N, Herregods SS, Verbruggen G, Herregods LL (2011). The effect of Lidocaine on viability of cultivated mature human cartilage cells: an in vitro study. Knee Surg Sports Traumatol Arthrosc..

[36] Grishko V, Xu M, Wilson G, Pearsall A. (2010). Apoptosis and mitochondrial dysfunction in human chondrocytes following exposure to lidocaine, bupivacaine, and ropivacaine. J Bone Joint Surg..

[37] Takeno K, Kobayashi S, Miyazaki T, Shimada S, Kubota M, Meir A (2009). Lidocaine cytotoxicity to the zygapophysial joints in rabbits: changes in cell viability and proteoglycan metabolism in Vitro. Spine..

[38] Bogatch MT, Ferachi DG, Kyle B, Popinchalk S, Howell MH, Ge D (2010). Is chemical incompatibility responsible for chondrocyte death induced by local anesthetics?. Am J Sports Med..

[39] Seshadri V, Coyle CH, Chu CR (2009). Lidocaine potentiates the Chondrotoxicity of methylprednisolone. Arthroscopy..

[40] Dragoo JL, Braun HJ, Kim HJ, Phan HD, Golish SR (2012). The in Vitro chondrotoxicity of single-dose local anesthetics. Am J Sports Med..

[41] Piat P, Richard H, Beauchamp G, Laverty S. (2012). In vivo effects of a single intra-articular injection of 2% lidocaine or 0,5% bupivacaine on articular cartilage of normal horses. Vet Surg..

[42] Olsen PC, Coelho LP, Costa JCS, Cordeiro RSB, Silva PMR, Martins MA (2012). Two for one: cyclic AMP mediates the anti-inflammatory and anti-spasmodic properties of the non-anesthetic lidocaine analog JMF2-1. Eur J Pharmacol..

[43] Gordon SM, Chuang BP, Wang XM, Hamza MA, Rowan JS, Brahim JS (2008). The differential effects of bupivacaine and lidocaine on prostaglandin E2 release, cyclooxygenase gene expression and pain in a clinical pain model. Anesth Analg..

[44] Raynauld JP, Buckland-Wright C, Ward R, Choquette D, Haraoui B, Martel-Pelletier J (2003). Safety and efficacy of long-term intra-articular steroid injections of the knee: a randomized, double-blind, placebo-controlled trial. Arthritis Rheum..

[45] McAlindon TE, LaValley MP, Harvey WF, Price LL, Driban JB, Zhang M (2017). Effect of intra-articular triamcinolone vs saline on knee cartilage volume and pain in patients with knee osteoarthritis – a randomized clinical trial. JAMA..

